# Acute Pain Management Following Mandibular Third Molar Exodontia: A Bibliometric Analysis of Randomized Controlled Trials

**DOI:** 10.1016/j.identj.2024.09.018

**Published:** 2024-10-06

**Authors:** Jinwei Huang, Yena Gan, He Xu, He Zhu, Sheng Han, Nan Li, Duoduo Li, Zhigang Cai

**Affiliations:** a Department of General Dentistry Ⅱ, Peking University School and Hospital of Stomatology, Beijing, P.R. China; b National Center for Stomatology & National Clinical Research Center for Oral Diseases & National Engineering Research Center of Oral Biomaterials and Digital Medical Devices, Beijing, P.R. China; c Department of Tuina and Pain, Dongzhimen Hospital, Beijing University of Chinese Medicine, Beijing, P.R. China; d Department of Pediatric Dentistry, Peking University School and Hospital of Stomatology, Beijing, P.R. China; e Department of Academic Research, International Research Center for Medicinal Administration, Peking University, Beijing, P.R. China; f Department of Epidemiology and Biostatistics, School of Public Health, Peking University Health Science Center, Beijing, P.R. China; g Department of Oral and Maxillofacial Surgery, Peking University School and Hospital of Stomatology, Beijing, P.R. China

**Keywords:** Analgesia, Bibliometric analysis, Exodontia, Mandibular third molar, Postoperative pain

## Abstract

**Introduction and aims:**

To reveal the evolution of pain management strategies following mandibular third molar (M3M) exodontia, examine the geographic contribution of research, and explore future developments through a bibliometric analysis.

**Methods:**

A comprehensive search was conducted in various leading databases. Data on bibliometrics, participant demographics, and agent regimens were extracted for eligible studies. Descriptive bibliometrics, citation analysis, and keyword bursts were performed to assess the research outputs, distribution, and emerging hotspots.

**Results:**

A total of 173 randomized control trials from 2004 to 2024 were included. The number of publications showed a consistent upward trend since 2007. Brazil exhibited the most publications and citations. Germany presented the highest mean citations per publication. Brazil, Spain, and Italy showed the closest collaboration. Appropriately 14,391participants with 14,710 extracted M3M were enrolled. Nonsteroidal anti-inflammatory drugs (NSAIDs) were the most extensively studied analgesics, followed by glucocorticoids, opioids, and paracetamol. NSAIDs and paracetamol were predominantly administered orally, whereas glucocorticoids and opioids were primarily applied topically (*P* < .001). Studies on opioids significantly predated the studies using other agents. Adverse events were found in 50.87% of the included studies, where nausea and vomiting were the most frequently reported. Tramadol and piroxicam have drawn increasing interest in recent years.

**Conclusions:**

This study revealed information on the research outputs, distribution, and future developments of analgesic agents following M3M exodontia. Brazil exhibited the highest level of productivity and recorded the most citations. NSAIDs generated the largest amount of research and are emerging as a benchmark for comparative studies. Oral administration is the most frequently used approach for agent delivery. Nausea and vomiting are the most commonly reported adverse effects.

**Clinical relevance:**

The bibliometric analysis offers insights into the field of pain management following mandibular 3rd molar exodontia and how it has evolved. Tramadol and piroxicam have become research hotspots in recent years.

## Introduction

Mandibular third molars (M3M) may commonly be accompanied by many pathologies, as caries, pericoronitis, dentition crowding, and adjacent tooth lesions, requiring timely exodontia.^1^, ^2^, ^3^ Approximately 10 million M3Ms are extracted in the United States of America annually.^4^ Subsequent moderate to severe postoperative pain compromises patients’ quality of life and adherence to succeeding treatments.^5^ Therefore, effective pain and swelling management is essential for proper patient care, as it may lead to systemic health morbidities.^6^, ^7^, ^8^

Postoperative pain is induced by algogenic substances generated from the periphery. The afferent activity is relayed to the dorsal horn neurons of the spinal cord and central nervous system (CNS).^9^ Multiple pharmaceutical agents have been developed to alleviate such pain, including nonsteroidal anti-inflammatory drugs (NSAIDs), glucocorticoids, paracetamols, opioids, and other novel agents. These vary in components, brands, package sizes, formulations, doses, and administration routes.^10^ Combined therapies of various agents have been advocated for because they may provide multimodal coverage of a wider spectrum of pain due to synergistic effects.^11^ There is considerable debate surrounding the most effective pain relief approaches following M3M exodontia because different drug delivery routes produce varying therapeutic outcomes. Oral administration of analgesic agents is widely recommended due to its convenience, rapid absorption, and high efficacy. However, ensuring patients’ adherence to the pharmaceutical instructions may be challenging, raising concerns about the strategies’ trueness.^12^ In contrast, topical administration has been proven effective and independent of the patient's adherence. Nevertheless, the optimal selection of drugs, dosage, and delivery approaches remains debatable.

Clinicians need to keep up-to-date with paradigms in pain management when making decisions.^13^ Previous studies have tended to focus on a specific category of agent, neglecting a comprehensive overview.^14^ Bibliometric analysis is an emerging tool to obtain crucial information from massive publications using statistical methods, which has been extensively used to provide scientific landscapes and disclose knowledge structures. To our knowledge, there is no previous analysis of the research outputs, distribution, and knowledge gaps on this topic. Therefore, this study aimed to conduct a thorough bibliometric analysis to examine the existing randomized controlled trials (RCTs) on the pharmacological management of postoperative pain following M3M exodontia. It is beneficial to reveal the evolution of pain management strategies, examine the geographic contribution of research, and explore future developments in this field. This study might contribute to a scientific framework for enhancing pain control and advocating better practices.

## Materials and methods

### Data sources and search strategy

A comprehensive search was conducted using the Web of Science Core Collection, Scopus, PubMed, Embase, and Cochrane Library databases in March 2024. The combination of keywords (‘third molar’, ‘mandibular’, ‘extraction/exodontia’, and ‘pain management’) or synonyms was searched in the titles, abstracts, or keywords (Supplemental Table 1). The identified papers were independently screened by two investigators to determine eligibility. Full texts were assessed in cases with insufficient or missing information. Discrepancies were resolved through discussion with a third investigator.

### Study selection and eligibility criteria

The study protocol was registered with the International Prospective Register of Systematic Reviews (PROSPERO, no. CRD42024572107). The PICOS principle was applied to identify eligible RCTs. The inclusion criteria were: (1) P: participants underwent M3M exodontia; (2) I: participants took various types of analgesic agents; (3) C: participants took placebo or other agents; (4) O: pain intensity (assessed by visual analog scale, VAS^15^ or numerical rating scale, NRS,^16^ facial oedema, mouth opening, rate of rescue medicine (RM) use, adverse events, and participant satisfaction; and (5) S: RCTs that were published in English between 2004 and 2024.

The exclusion criteria were: (1) publications before 2004; (2) animal studies; (3) letters, commentaries, and conference abstracts; (4) studies involving nonpharmacological therapies, such as laser therapy, acupuncture, and aromatherapy; (5) cross-sectional studies, case series, and reviews; and (6) exodontia other than M3M.

### Data extraction

A data cleaning process was conducted before data extraction to improve accuracy; for instance, keywords without substantial meaning were removed (eg, articles). The following information was extracted using a previously designed Microsoft Excel spreadsheet: (1) year of publication, authors, country, affiliation, title, journal, Journal Citation Reports division, impact factor (IF), keywords, citations, and references; (2) participant demographics: sample size, sex, age, American Society of Anesthesiology status,^17^ and Pell and Gregory classification of M3M^18^; (3) agent regimens: agent type, dosage, route and frequency of administration, delivery timing, and follow-up duration; and (4) outcome indicators: pain intensity (VAS or NRS), facial oedema, mouth opening, participant satisfaction, follow up, time to first use of rescue medications, and adverse events.

### Statistical analysis

**Fig. 1:**
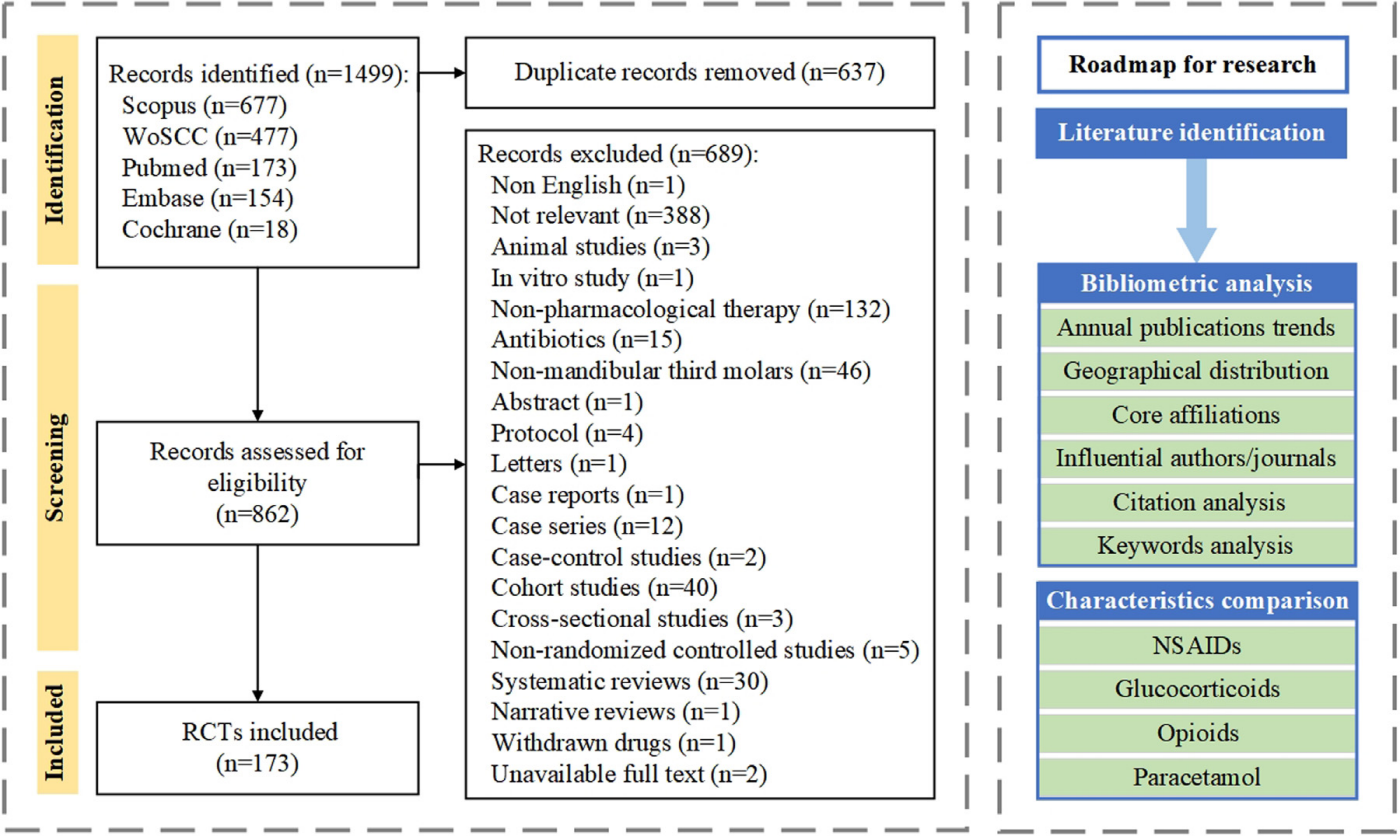
Flowchart diagram of study selection and research methodology.

Descriptive bibliometrics, citation analysis, and keyword bursts were performed to assess the annual and global distribution of RCTs, as well as influential journals, countries, affiliations, and topic trends. The mapping of the bibliographic data was performed using the bibliometrix and ggplot packages of the R software (version 4.3.1; R Foundation for Statistical Computing). Descriptive statistics were performed for various types of analgesics to examine various variables. Continuous variables were summarized using mean ± standard deviation, where analysis of variance and multiple comparison methods such as Games-Howell test were employed to compare the differences in distributions. The number of extractions, sex distribution, route of administration, and delivery timing were analysed using the Chi-squared test. All statistical analyses were conducted with the significance level set at *P* < .05. The flowchart illustrates the study selection process and research methodology (Figure 1).

## Results

**Fig. 2:**
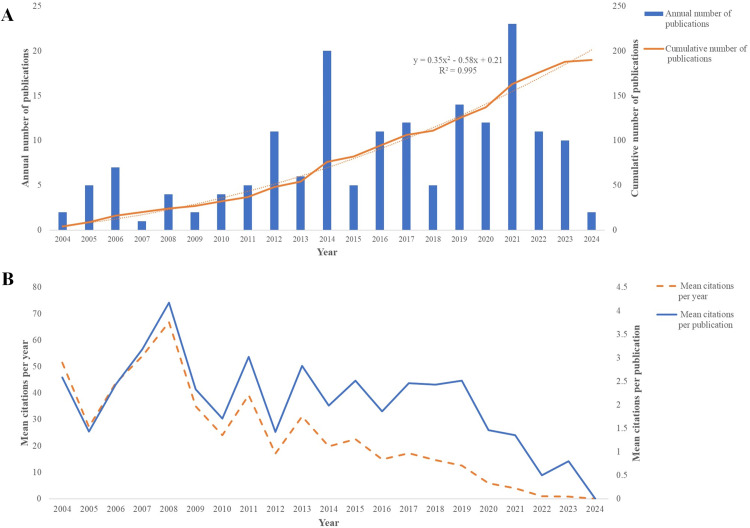
Global trends of RCTs on pharmacological management of postoperative pain following M3M exodontia. (A) Annual and accumulated number of publications. (B) Mean citations. M3M, mandibular third molars; RCT, randomized controlled trials.

A total of 173 RCTs published from 2004 to 2024 across 74 journals were included, which were coauthored by 737 researchers from 40 countries. The number of publications has shown a consistent upward trend since 2007, growing at an average annual rate of 2.93%. The cumulative publications follow an exponential curve ($y\;=\;0.35x^{2}-0.58x+0.21$, $R^{2}\;=\;$0.995), indicating that the number of publications is likely to continue increasing (Figure 2). Most of the included RCTs have an average annual citation rate ranging from 1 to 4 times. After data cleaning, a total of 186 unique keywords were obtained. Tramadol and piroxicam have drawn increasing interest from researchers in the past 5 years.

**Fig. 3:**
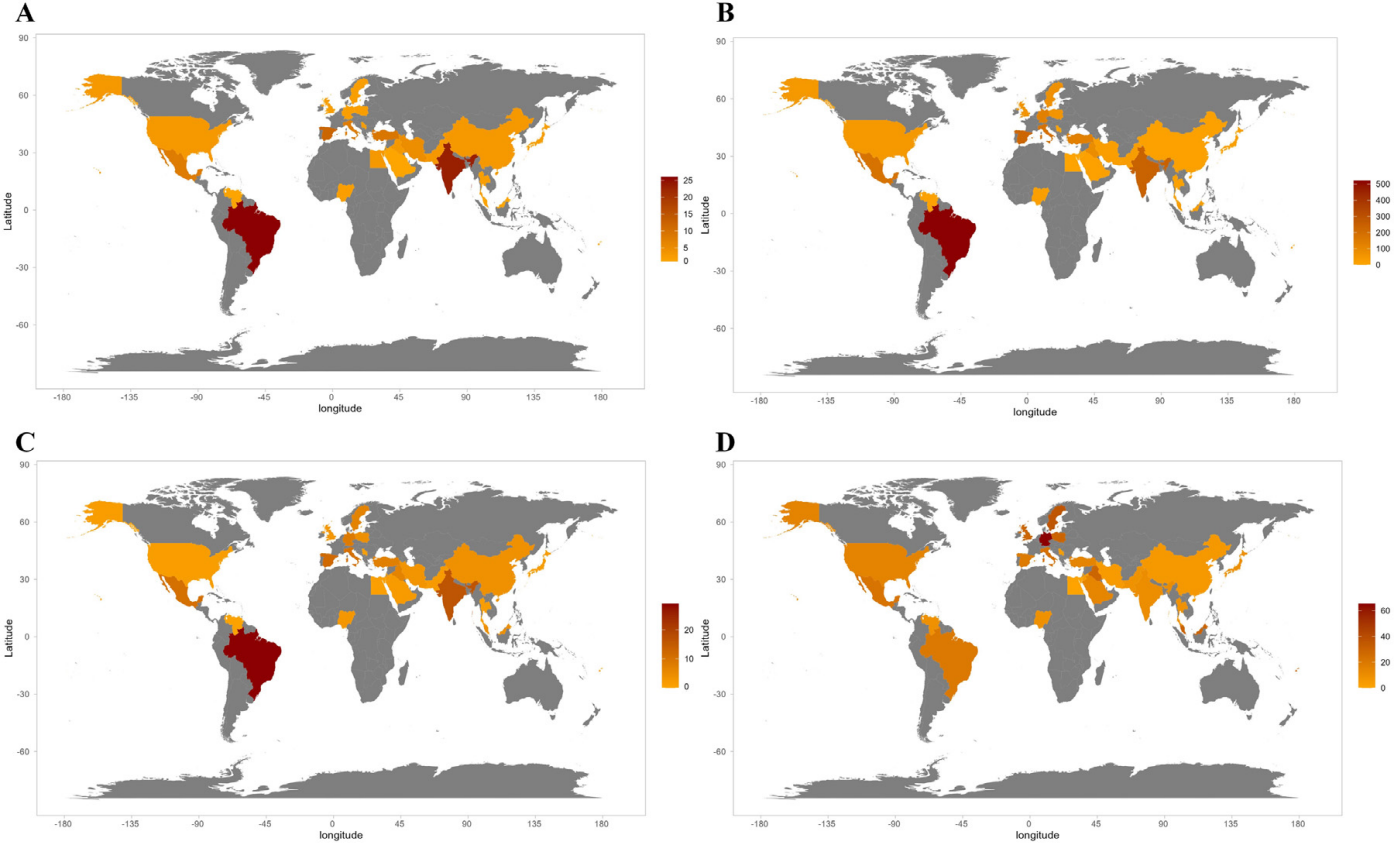
The geographic contribution of countries based on corresponding authors. (A) Overall publications; (B) overall citations; (C) mean citations per year; (D) mean citations per publication.

**Fig. 4:**
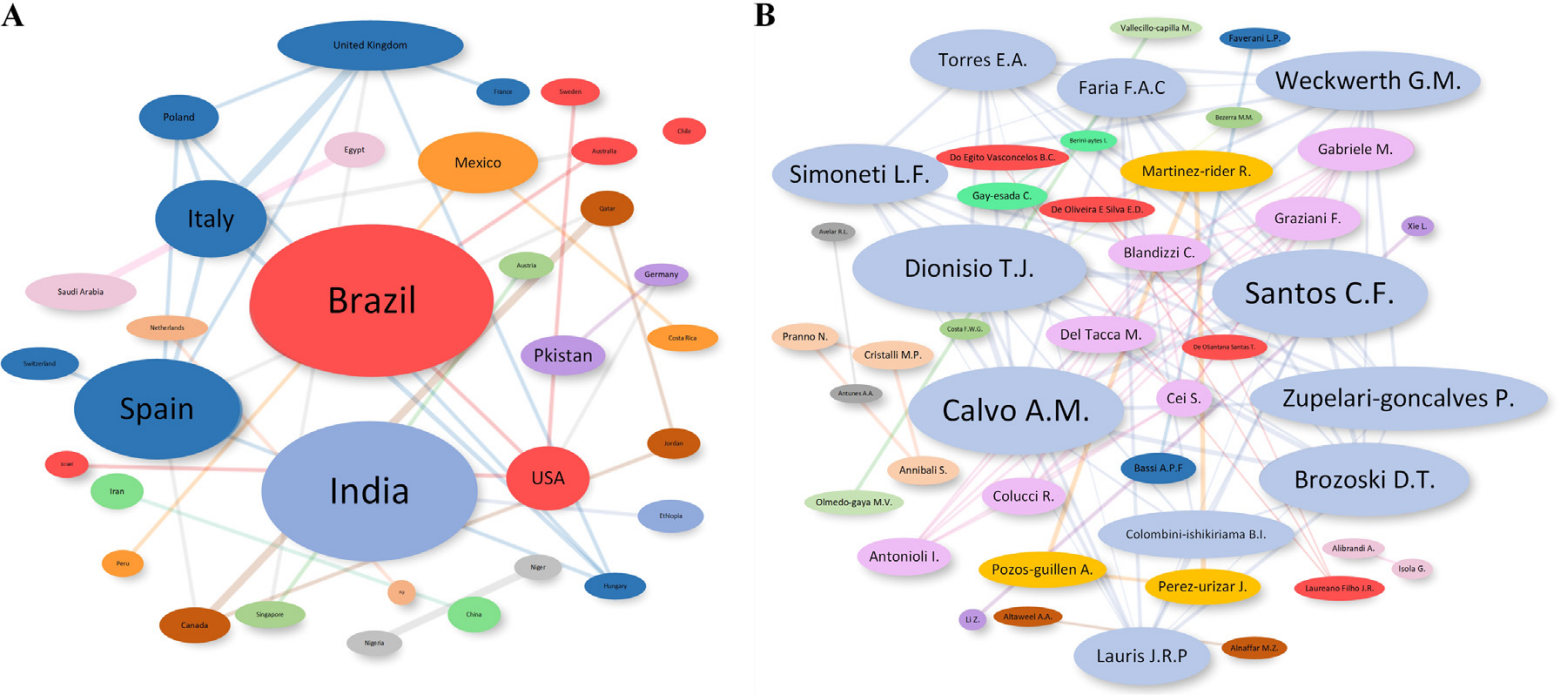
Collaboration networks. (A) Country collaborations. (B) Author collaborations.

Brazil has led the way in terms of published RCTs (15.03%), followed by India (13.29%) and Spain (6.94%). Brazil also received the highest number of 522 citations and annual mean citations (29.00 citations per year) (Table 1). Nevertheless, Germany presented the highest mean citations per publication at 65.50 (Figure 3). Among the top 10 countries in terms of publications and citations, Brazil, Spain, and Italy showed the closest collaboration (Figure 4).

**Table 1:** Most productive and influential countries, authors, and journals.

Rank	Country productivity (publications)	Country influence (citations)	Author productivity (publications)	Author influence (citations)	Journal productivity (publications)	Journal influence (citations)
1	Brazil (26)	Brazil (522)	Bassi A.P.F. (5)	Gabriele M. (157)	Journal of Oral and Maxillofacial Surgery (27)	International Journal of Oral and Maxillofacial Surgery (1109)
2	India (23)	India (243)	Calvo A.M. (5)	Graziani F. (157)	International Journal of Oral and Maxillofacial Surgery (24)	Medicina Oral Patologia Oral Y Cirugia Bucal (369)
3	Spain (12)	Spain (225)	Dionisio T.J. (5)	Gay-Escoda C. (152)	Medicina Oral Patologia Oral Y Cirugia Bucal (22)	Journal of Oral and Maxillofacial Surgery (366)
4	Turkey (9)	Italy (186)	Gay-Escoda C. (5)	Laureano Filho J.R. (150)	Clinical Oral Investigations (5)	Anesthesia And Analgesia (107)
5	Mexico (8)	Mexico (178)	Martinez-Rider R. (5)	Majid O.W. (137)	Journal of Evidence-Based Dental Practice (4)	Oral Surgery, Oral Medicine, Oral Pathology and Oral Radiology (95)
6	Italy (7)	Turkey (131)	Santos C.F. (5)	Tonelli M. (132)	Oral Surgery, Oral Medicine, Oral Pathology and Oral Radiology (4)	British Journal of Oral and Maxillofacial Surgery (93)
7	Iran (5)	Germany (131)	Brozoski D.T. (4)	Martinez-Rider R. (122)	British Journal of Oral and Maxillofacial Surgery (3)	Journal of Cranio-Maxillofacial Surgery (89)
8	China (3)	Singapore (107)	Perez-Urizar J. (4)	Arduino P.G. (121)	Journal of Cranio-Maxillofacial Surgery (3)	Pain (87)
9	Egypt (3)	Iraq (86)	Pozos-Guillen A. (4)	Daiuti F. (121)	Anesthesia And Analgesia (2)	European Journal of Clinical Pharmacology (43)
10	Iraq (3)	Sweden (72)	Annibali S. (3)	ALLAIS M. (112)	European Journal of Clinical Pharmacology (2)	British Journal of Anaesthesia (42)

Three journals published approximately one-third of the included RCTs, including the *Journal of Oral and Maxillofacial Surgery* (*n* = 27, 15.61%, IF = 1.9, 2022), *International Journal of Oral and Maxillofacial Surgery* (*n* = 24, 13.87%, IF = 2.4, 2022), and *Medicina Oral Patologia Oral Y Cirugia Bucal* (*n* = 22, 12.72%, IF = 2.2, 2022). They were considered core journals in this field. The included 173 RCTs were cited 2914 times (ranging from 0 to 121), with an average of 16.84. Notably, citations from the core journals were significantly higher than those of other journals. Approximately 28.90% of the RCTs had over 20 citations (Supplemental Table 2), with 56.00% of them being published in these core journals.

**Fig. 5:**
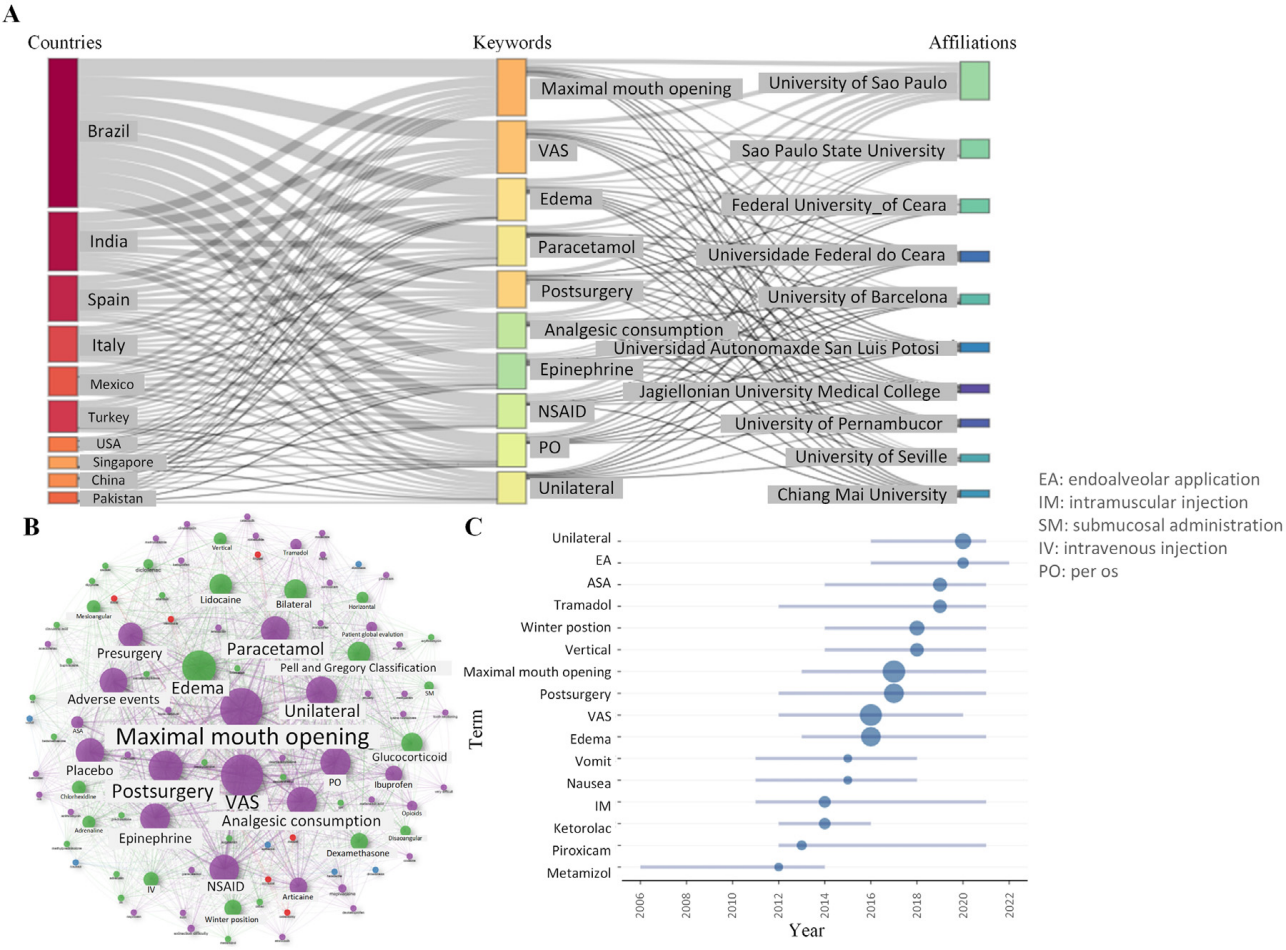
Visualization of keywords. (A) Three-field plot among countries, affiliations, and keywords. (B) Word-cloud Network generated by the walktrap algorithm. (C) Trend topics with occurrences≥6. The horizontal lines and the nodes indicate the time duration and the median time that the keywords appeared, respectively.

A total of 14,391 participants aged 18 to 35 years were involved, with a record of 14,710 M3M extracted. Out of these participants, the sex of 1370 individuals was not disclosed. However, 5927 males and 7094 females were identified. Most of the extracted M3M were categorized as Pell and Gregory I to II and A to B, and the participants’ physical status was rated American Society of Anesthesiology I to II. The Winter's position was also used to describe the impaction status of the M3Ms in 11.42% (1680/14,710) of the cases. Among these cases, mesioangular impaction (33.10%) was the most common type, followed by horizontal (31.85%), vertical (25.36%), and distoangular (9.70%) (Figure 5). Majority of the participants underwent unilateral M3M extraction. According to the description of the included studies, flap surgeries were required in at least 51.34% (7388/14,391) of the cases.

The analgesic agents were primarily delivered through oral administration (57.80%), followed by intravenous injection (23.12%), submucosal administration (15.03%), intraalveolar administration (10.40%), intramuscular injection (10.40%), and sublingual administration (2.31%). Up to 56.10% of the included RCTs focused on postoperative administration of analgesics, while 41.46% investigated preoperative administration and 2.44% examined both pre- and postoperative administration (Figure 5). NSAIDs were the most extensively studied analgesics among all included RCTs (58.38%), followed by glucocorticoids (36.99%), opioids (19.08%), and paracetamol (8.09%). Ibuprofen was the most commonly chosen NSAIDs (26.73%), followed by diclofenac (19.80%) and ketorolac (8.91%). Dexamethasone was the most frequently selected glucocorticoid (71.88%), followed by methylprednisolone (21.88%), prednisolone (6.25%), and betamethasone (4.69%). Tramadol was the most frequently selected opioid (66.67%), followed by codeine (15.15%), bupivacaine (9.09%), morphine (6.06%), and tapentadol (3.03%). Approximately 53.18% of the included studies employed a placebo for control purposes. The use of ibuprofen and tramadol for postoperative pain relief has been a research hotspot over the past 5 years.

Up to 93.64% of the RCTs employed patient-reported outcomes of VAS to assess the intensity of pain, while only a few studies adopted the NRS.^15^^,^^16^^,^^19^, ^20^, ^21^, ^22^, ^23^ Additionally, many other outcomes were also reported because of their clinical significance. Approximately 69.36% of the RCTs measured the maximum mouth opening, 66.47% examined facial oedema, and 16.18% assessed participant satisfaction. The use of RM was reported in 72.25% of the included RCTs, where paracetamol was the most commonly used (58.40%), followed by ibuprofen (16.00%) and codeine (5.60%). Adverse events were found in 50.87% of the included RCTs, where nausea and vomiting were the most frequently reported (Figure 5).

A significant difference was observed in the ‘route of administration’ of NSAIDs, glucocorticoids, opioids, and paracetamol (*P* < .001). NSAIDs and paracetamol were predominantly administered orally, whereas glucocorticoids and opioids were primarily applied topically. Studies using opioids as analgesics significantly predated the studies using the other three agents. No significant differences were found in terms of citations, participant age, sex, operating time, time to first use of RM, and delivery timing (Table 2).

**Table 2:** Difference of research characteristics among NSAIDs, glucocorticoids, opioids, and paracetamol.

Category	NSAIDs	Glucocorticoids	Opioids	Paracetamol	*P* value
Citations	16.13 ± 17.96	20.38 ± 24.81	16.36 ± 18.65	16.32 ± 19.82	.585
Age (y)	25.89 ± 0.31	24.10 ± 0.19	23.86 ± 0.20	27.92 ± 0.13	.341
No. of extractions (M/F)	4758 (2089/2641)	2079 (924/1062)	1127 (517/610)	363 (160/203)	.299
Operating time (min)	20.29 ± 1.06	26.34 ± 0.41	23.80 ± 2.97	18.83 ± 0.32	.178
Time to first use of rescue medication (min)	415.05 ± 0.97	-	285.60 ± 0.12	271.09 ± 0.53	.852
Delivery timing					.851
Postoperative	54	37	10	4	
Preoperative	7	4			
Pre- and postoperative	88	59	22	6	
Route of administration					<.001*
Topical	119	21	13	8	
Oral	30	79	19	2	

d, day; F, female; M, male; NSAIDs, nonsteroidal anti-inflammatory drugs; y, years.

⁎ *P* < .05.

## Discussion

Because of the high predictability of a well-defined trajectory, M3M exodontia has become an excellent acute pain model to facilitate the evaluation of analgesic effectiveness.^24^ This model has contributed to understanding the multifactorial phenomenon of pain and minimized the confounding factors associated with other models.^25^ The current study suggests that there has been an extensive international focus on investigating the analgesic efficacy of various pharmacological agents following M3M exodontia over the past two decades, which is also expected to continue and intensify in the future. Notably, researchers from Brazil, India, and Spain have made significant contributions in this field in terms of publications and citations. Brazil, in particular, places great importance on public health and has implemented various programs to enhance public awareness and accessibility to dental services.^26^ The findings also showed that the selection of analgesics might influence the guidelines endorsed by different countries. The extensive research output of Brazil indicates that its guidelines heavily rely on the thoroughly studied agents of NSAIDs and paracetamol. With the exponential growth of research, there has been a corresponding increase in the prescription rate of these analgesics in Brazil.^27^ However, in the US guideline, opioids remain an essential part of pain management despite the risk of addiction.^28^ The current study also reveals an increasing research interest in tramadol and piroxicam, suggesting ongoing efforts to balance effective pain relief and safety concerns.

The publication timing and citation patterns of these studies demonstrated a shifting of researcher's concerns. Specifically, a notable increase in focus on glucocorticoids and opioids was observed in the highly-cited RCTs (over 20 citations) published between 2007 and 2012, while the concern for NSAIDs declined. Furthermore, a continued rise in interest in glucocorticoids and paracetamol was found in the highly-cited RCTs published between 2013 and 2018. Although controversial, citation counts help researchers identify landmark studies. The top-cited studies of the present study exhibited high-quality RCT designs and scientific findings.

NSAIDs are widely and preferentially used in clinical practice, which inhibit the release of cyclooxygenase enzymes (COX-1 and COX-2) and decrease the production of pain-related prostaglandins within the injured tissue.^5^^,^^29^ Among the various NSAIDs, ibuprofen (400 mg) frequently serves as the referred standard or baseline.^14^^,^^30^ Piroxicam is known for its long-lasting analgesic effects,^31^ while celecoxib has minimal gastrointestinal side effects.^32^ These two agents have been considered the hotspot of NSAIDs for the past 5 years, which generated the most studies. Glucocorticoids are also used because of their substantial anti-inflammatory effects.^33^ They act in the initial phase of inflammation by inhibiting phospholipase A2 and reducing arachidonic acid, thereby reducing the inflammatory response. Dexamethasone and methylprednisolone are widely used.^34^ Paracetamol is another widely used non-narcotic analgesic. Current evidence suggests that the analgesic effect occurs mainly by activating specific serotonin pathways and inhibiting prostaglandins.^35^ Opioids act in the CNS through μ-opioid receptors but do not provide any anti-inflammatory action^36^ and are less used because of the side effects.^30^ Nevertheless, the opioid epidemic has ushered in a new era of provider-directed analgesic therapy in dentistry.^13^ A multimodal analgesia combination seemed to provide better benefits since it delivers sufficient pain relief through synergistic effects, with a concomitant reduction of side effects owing to lower doses of individual drugs.^37^^,^^38^

The analgesic agents could be delivered through various approaches. The NSAIDs and paracetamol were typically delivered orally to provide sustained blood levels for long-term management.^39^ Based on the current evidence of literature, they were generally recommended to manage mild to moderate pain for the majority of patients. For those who experienced general discomfort as a result of systemic use of analgesics or when oral administration was not sufficiently rapid to achieve the desired therapeutic effect, topical administration can serve as a supplementary alternative. For instance, glucocorticoids, as well as opioids, could also be administered through submucosal, intramuscular, and intraalveolar routes.^40^ Submucosal administration allowed for rapid onset of action by bypassing the enterohepatic circulation, while intramuscular injection ensured faster absorption and longer duration of action.^41^ Intraalveolar administration (also named endoalveolar administration) was preferred to provide a rapid localized therapeutic effect where the analgesic dressings were applied incrementally into the extraction socket.^42^^,^^43^

According to the available evidence and identified trends, varying patterns in the utilization of analgesics were emphasized. Corticosteroids are mainly administered topically to alleviate moderate to severe pain or when oral intake is not viable. Dexamethasone effectively reduces inflammation in specific areas and has become a hotspot for research. Opioids are primarily used to alleviate acute severe pain when given intramuscularly or topically. Tramadol, the most commonly used opioid in the past 5 years, has potent analgesic effects. However, there has been restricted use in recent years due to the opioid epidemic. Instead, a shift to multimodal combined analgesia is noticed to increase. Besides, an individualized analgesic strategy is also suggested to ensure optimal pain relief considering patient-specific factors.^44^, ^45^, ^46^

The timing of administration appears to affect the effectiveness of the analgesics. Preemptive therapies seem to mitigate postoperative nociception to the CNS and provide superior benefits.^13^ NSAIDs, when administered preoperatively, can be rapidly absorbed and distributed to oral tissues before the initiation of inflammation. This was confirmed in the present study by observing the effects of naproxen, nimesulide, celecoxib, ibuprofen, rofecoxib, and etoricoxib. They usually reach a peak plasma concentration after 2 hours of drug administration and function for up to 6 hours.^47^ Therefore, the timing of analgesic administration of other agents is an area of interest for future research.^48^

This is the first bibliometric analysis in the pharmacological management of postoperative pain following M3M extraction. However, this study has inherent limitations. First, although leading databases were incorporated as data sources, high-quality literature from other databases may have been omitted. Second, only English publications were included, which might have introduced a selection bias. Third, this study evaluated the academic influence through citation analysis instead of examining the quality of the implementation of the included RCTs, consequently, the findings obtained might not be fully robust. Lastly, this study identified the crucial analgesic agents for M3M extractions, as well as their administration routes and timing. However, the limited amount of literature and numerous bias factors hindered further analysis of the effectiveness of the studied agents. A well-designed Meta-analysis is warranted for developing optimal analgesic strategies. Therefore, the outcomes obtained should be interpreted with caution.

## Conclusions

This study revealed information on the research outputs, distribution, and future developments of pharmacological agents for managing postoperative pain following M3M exodontia, including NSAIDs, glucocorticoids, paracetamols, and opioids. Brazil exhibited the highest level of productivity in conducting RCTs and recorded the highest number of citations. NSAIDs generated the largest amount of research and are emerging as a benchmark for comparative studies. Oral administration is the most frequently used approach for delivering analgesic agents, particularly postoperatively. Nausea and vomiting are the most commonly reported adverse effects.

## Conflict of interest

None disclosed.

## Appendix Supplementary materials

Image, application 1

Image, application 2

## Footnotes

Supplementary material associated with this article can be found in the online version at doi:10.1016/j.identj.2024.09.018.
