# Supplementary material for: Acute Pain Management Following Mandibular Third Molar Exodontia: A Bibliometric Analysis of Randomized Controlled Trials
Source: Int Dent J. 2024 Oct 6;75(2):939–48. doi: 10.1016/j.identj.2024.09.018 (PMC11976606; doi:10.1016/j.identj.2024.09.018)
Supplement: Supplementary file 1 [file mmc1.docx]

**Supplemental Table 1 - Data sources, search strategy, and outcomes**.

| Database | Search strategy | Results  (2024-03-21) |
| --- | --- | --- |
| Scopus | #1 TITLE-ABS-KEY = ((third molar) OR (wisdom tooth) OR (wisdom teeth))  #2 TITLE-ABS-KEY = (mandibular OR mandible OR lower)  #3 TITLE-ABS-KEY = (extraction OR removal OR exodontia)  #4 TITLE-ABS-KEY = (analgesia OR painkiller OR (pain management) OR (pain control))  #5 LANGUAGE = (English)  #6 DOCTYPE = (Article)  #7 YEAR from 2004 to 2024  #8 #1 AND #2 AND #3 AND #4 AND #5 AND #6 AND #7 | 697 |
| Web of Science Core Collection (SCI-EXPANDED) | #1 TS = ((third molar) OR (wisdom tooth) OR (wisdom teeth))  #2 TS = (mandibular OR mandible OR lower)  #3 TS = (extraction OR removal OR exodontia)  #4 TS = (analgesia OR painkiller OR (pain management) OR (pain control))  #5 LA = (English)  #6 DT = (Article)  #7 PY = (2004 OR 2005 OR 2006 OR 2007 OR 2008 OR 2009 OR 2010 OR 2011 OR 2012 OR 2013 OR 2014 OR 2015 OR 2016 OR 2017 OR 2018 OR 2019 OR 2020 OR 2021 OR 2022 OR 2023 OR 2024)  #8 #1 AND #2 AND #3 AND #4 AND #5 AND #6 AND #7 | 497 |
| PubMed (MEDLINE) | #1 "third molar"[Title/Abstract] OR "wisdom tooth"[Title/Abstract] OR "wisdom teeth"[Title/Abstract]  #2 "mandibular"[Title/Abstract] OR "mandible"[Title/Abstract] OR "lower"[Title/Abstract]  #3 "extraction"[Title/Abstract] OR "removal"[Title/Abstract] OR "exodontia"[Title/Abstract]  #4 "analgesia"[Title/Abstract] OR "painkiller"[Title/Abstract] OR "pain management"[Title/Abstract] OR "pain control"[Title/Abstract]  #5 2004/1/1:2024/1/23[pdat]  #6 English[Language]  #7 #1 AND #2 AND #3 AND #4 AND #5 AND #6 | 179 |
| CENTRAL (The Cochrane Library) | #1 ((third molar) OR (wisdom tooth) OR (wisdom teeth)):ti,ab,kw  #2 (mandibular OR mandible OR lower):ti,ab,kw  #3 (extraction OR removal OR exodontia):ti,ab,kw  #4 (analgesia OR painkiller OR (pain management) OR (pain control)):ti,ab,kw  #5 publication date from Jan 2004 to Jan 2024  #7 #1 AND #2 AND #3 AND #4 AND #5 AND #6 | 18 |
| Embase | #1 'third molar':ti,ab,kw OR 'wisdom tooth':ti,ab,kw OR 'wisdom teeth':ti,ab,kw  #2 mandibular:ti,ab,kw OR mandible:ti,ab,kw OR lower:ti,ab,kw  #3 extraction:ti,ab,kw OR removal:ti,ab,kw OR exodontia:ti,ab,kw  #4 analgesia:ti,ab,kw OR painkiller:ti,ab,kw OR 'pain management':ti,ab,kw OR 'pain control':ti,ab,kw  #5 [english]/lim  #6 [article]/lim  #7 [2004-2024]/py  #8 #1 AND #2 AND #3 AND #4 AND #5 AND #6 AND #7 | 161 |
