# Supplementary material for: Acute Pain Management Following Mandibular Third Molar Exodontia: A Bibliometric Analysis of Randomized Controlled Trials
Source: Int Dent J. 2024 Oct 6;75(2):939–48. doi: 10.1016/j.identj.2024.09.018 (PMC11976606; doi:10.1016/j.identj.2024.09.018)
Supplement: Supplementary file 2 [file mmc2.docx]

**Supplemental Table 2 - Top 50 highly-cited studies**.

| Study | Title | Journal | Citations |
| --- | --- | --- | --- |
| Graziani F., 2006 | Perioperative dexamethasone reduces post-surgical sequelae of wisdom tooth removal. A split-mouth randomized double-masked clinical trial | International Journal of Oral and Maxillofacial Surgery | 121 |
| Laureano Filho J.R., 2008 | Clinical comparative study of the effectiveness of two dosages of Dexamethasone to control postoperative swelling, trismus and pain after the surgical extraction of mandibular impacted third molars | Medicina Oral, Patologia Oral Y Cirugia Bucal | 112 |
| Kleinert R., 2008 | Single dose analgesic efficacy of tapentadol in postsurgical dental pain: The results of a randomized, double-blind, placebo-controlled study | Anesthesia and Analgesia | 83 |
| Buyukkurt M.C., 2006 | The Effect of a Single Dose Prednisolone With and Without Diclofenac on Pain, Trismus, and Swelling After Removal of Mandibular Third Molars | Journal of Oral and Maxillofacial Surgery | 78 |
| Quiding H., 2013 | TRPV1 antagonistic analgesic effect: A randomized study of AZD1386 in pain after third molar extraction | Pain | 67 |
| Antunes A.A., 2011 | Effect of two routes of administration of dexamethasone on pain, edema, and trismus in impacted lower third molar surgery | Oral and Maxillofacial Surgery | 66 |
| Majid O.W., 2011 | Effect of submucosal and intramuscular dexamethasone on postoperative sequelae after third molar surgery: Comparative study | British Journal of Oral and Maxillofacial Surgery | 64 |
| Isola G., 2019 | Evaluation of the efficacy of celecoxib and ibuprofen on postoperative pain, swelling, and mouth opening after surgical removal of impacted third molars: a randomized, controlled clinical trial | International Journal of Oral and Maxillofacial Surgery | 63 |
| Ong C.K.S., 2004 | Preoperative ketorolac has a preemptive effect for postoperative third molar surgical pain | International Journal of Oral and Maxillofacial Surgery | 61 |
| Chopra D., 2009 | A randomized, double-blind, placebo-controlled study comparing the efficacy and safety of paracetamol, serratiopeptidase, ibuprofen and betamethasone using the dental impaction pain model | International Journal of Oral and Maxillofacial Surgery | 58 |
| Pozos-Guillen A., 2007 | Pre-Emptive Analgesic Effect of Tramadol After Mandibular Third Molar Extraction: A Pilot Study | Journal of Oral and Maxillofacial Surgery | 54 |
| Koray M., 2014 | Efficacy of hyaluronic acid spray on swelling, pain, and trismus after surgical extraction of impacted mandibular third molars | International Journal of Oral and Maxillofacial Surgery | 51 |
| Majid O.W., 2014 | Perioperative Bromelain Reduces Pain and Swelling and Improves Quality of Life Measures After Mandibular Third Molar Surgery: A Randomized, Double-Blind, Placebo-Controlled Clinical Trial | Journal of Oral and Maxillofacial Surgery | 51 |
| Vegas-Bustamante E., 2008 | Efficacy of methylprednisolone injected into the masseter muscle following the surgical extraction of impacted lower third molars | International Journal of Oral and Maxillofacial Surgery | 48 |
| Rana M., 2013 | Evaluation of postoperative discomfort following third molar surgery using submucosal dexamethasone - A randomized observer blind prospective study | Oral Surgery, Oral Medicine, Oral Pathology and Oral Radiology | 48 |
| Ong C.K.S., 2005 | The analgesic efficacy of intravenous versus oral tramadol for preventing postoperative pain after third molar surgery | Journal of Oral and Maxillofacial Surgery | 46 |
| Jackson I.D., 2004 | Double-blind, randomized, placebo-controlled trial comparing rofecoxib with dexketoprofen trometamol in surgical dentistry | British Journal of Anaesthesia | 42 |
| Mico-Llorens J.M., 2006 | Efficacy of methylprednisolone in controlling complications after impacted lower third molar surgical extraction | European Journal of Clinical Pharmacology | 41 |
| Jung Y.-S., 2005 | The effects on postoperative oral surgery pain by varying NSAID administration times: Comparison on effect of preemptive analgesia | Oral Surgery, Oral Medicine, Oral Pathology, Oral Radiology and Endodontology | 37 |
| Aznar-Arasa L., 2012 | Effect of preoperative ibuprofen on pain and swelling after lower third molar removal: A randomized controlled trial | International Journal of Oral and Maxillofacial Surgery | 37 |
| Chugh A., 2018 | Submucosal injection of dexamethasone and methylprednisolone for the control of postoperative sequelae after third molar surgery: randomized controlled trial | International Journal of Oral and Maxillofacial Surgery | 37 |
| Albuquerque A.F.M., 2017 | Effect of pre-emptive analgesia on clinical parameters and tissue levels of TNF-α and IL-1β in third molar surgery: a triple-blind, randomized, placebo-controlled study | International Journal of Oral and Maxillofacial Surgery | 36 |
| Boonsiriseth K., 2012 | Comparative study of the effect of dexamethasone injection and consumption in lower third molar surgery | International Journal of Oral and Maxillofacial Surgery | 35 |
| Bhargava D., 2014 | Effects of intra-space injection of Twin mix versus intraoral-submucosal, intramuscular, intravenous and per-oral administration of dexamethasone on post-operative sequelae after mandibular impacted third molar surgery: a preliminary clinical comparative study | Oral and maxillofacial surgery | 34 |
| Kaczmarzyk T., 2010 | Preemptive effect of ketoprofen on postoperative pain following third molar surgery. A prospective, randomized, double-blinded clinical trial | International Journal of Oral and Maxillofacial Surgery | 32 |
| Costa F.W.G., 2015 | A split-mouth, randomized, triple-blind, placebo-controlled study to analyze the pre-emptive effect of etoricoxib 120 mg on inflammatory events following removal of unerupted mandibular third molars | International Journal of Oral and Maxillofacial Surgery | 32 |
| Isola G., 2019 | Analysis of the effectiveness of lornoxicam and flurbiprofen on management of pain and sequelae following third molar surgery: A randomized, controlled, clinical trial | Journal of Clinical Medicine | 32 |
| Madrazo-Jimenez M., 2016 | The effects of a topical gel containing chitosan, 0,2% chlorhexidine, allantoin and despanthenol on the wound healing process subsequent to impacted lower third molar extraction | Medicina Oral, Patologia Oral Y Cirugia Bucal | 31 |
| Morse Z., 2006 | Ibuprofen as a pre-emptive analgesic is as effective as rofecoxib for mandibular third molar surgery | Odontology | 30 |
| Simone J.L., 2013 | Comparative analysis of preemptive analgesic effect of dexamethasone and diclofenac following third molar surgery | Brazilian Oral Research | 30 |
| Lim D., 2017 | A Comparative Study on the Efficacy of Submucosal Injection of Dexamethasone Versus Methylprednisolone in Reducing Postoperative Sequelae After Third Molar Surgery | Journal of Oral and Maxillofacial Surgery | 29 |
| Da Costa Araujo F.A., 2012 | Comparative analysis of preemptive analgesic effect of tramadol chlorhydrate and nimesulide following third molar surgery | Journal of Cranio-Maxillofacial Surgery | 28 |
| Barbalho J.C., 2017 | Effects of co-administered dexamethasone and nimesulide on pain, swelling, and trismus following third molar surgery: a randomized, triple-blind, controlled clinical trial | International Journal of Oral and Maxillofacial Surgery | 28 |
| Gopalraju P., 2014 | Comparative study of intravenous Tramadol versus Ketorolac for preventing postoperative pain after third molar surgery - A prospective randomized study | Journal of Cranio-Maxillofacial Surgery | 27 |
| Moore R.A., 2015 | Validating speed of onset as a key component of good analgesic response in acute pain | European Journal of Pain | 27 |
| De Menezes S.A.F., 2010 | Efficacy of nimesulide versus meloxicam in the control of pain, swelling and trismus following extraction of impacted lower third molar | International Journal of Oral and Maxillofacial Surgery | 26 |
| Akbulut N., 2014 | Comparison of the effect of naproxen, etodolac and diclofenac on postoperative sequels following third molar surgery: A randomised, double-blind, crossover study | Medicina Oral Patologia Oral Y Cirugia Bucal | 26 |
| Mojsa I.M., 2017 | Effect of submucosal dexamethasone injection on postoperative pain, oedema, and trismus following mandibular third molar surgery: a prospective, randomized, double-blind clinical trial | International Journal of Oral and Maxillofacial Surgery | 26 |
| Graziani F., 2005 | Clinical evaluation of piroxicam-FDDF and azithromycin in the prevention of complications associated with impacted lower third molar extraction | Pharmacological Research | 25 |
| Isiordia-Espinoza M.A., 2011 | Preemptive analgesic effectiveness of oral ketorolac plus local tramadol after impacted mandibular third molar surgery | Medicina Oral, Patologia Oral Y Cirugia Bucal | 25 |
| de la Barrera-Nunez M.D., 2014 | Prospective double-blind clinical trial evaluating the effectiveness of bromelain in the third molar extraction postoperative period | Medicina Oral Patologia Oral Y Cirugia Bucal | 25 |
| Christensen K.S., 2008 | The analgesic efficacy and safety of a novel intranasal morphine formulation (morphine plus chitosan), immediate release oral morphine, intravenous morphine, and placebo in a postsurgical dental pain model | Anesthesia and Analgesia | 24 |
| Chaudhary P.D., 2015 | Pre-emptive effect of dexamethasone injection and consumption on post-operative swelling, pain, and trismus after third molar surgery. A prospective, double blind and randomized study | Journal of Oral Biology and Craniofacial Research | 24 |
| Orozco-Solis M., 2016 | Single dose of diclofenac or meloxicam for control of pain, facial swelling, and trismus in oral surgery | Medicina Oral, Patologia Oral Y Cirugia Bucal | 24 |
| Ceccheti M.M., 2014 | Analgesic and adjuvant anesthetic effect of submucosal tramadol after mandibular third molar surgery | Oral Surgery, Oral Medicine, Oral Pathology and Oral Radiology | 23 |
| Ehsan A., 2014 | Effects of pre-operative submucosal dexamethasone injection on the postoperative swelling and trismus following surgical extraction of mandibular third molar | Journal of the College of Physicians and Surgeons Pakistan | 23 |
| KoCer G., 2014 | Effect of the route of administration of methylprednisolone on oedema and trismus in impacted lower third molar surgery | International Journal of Oral and Maxillofacial Surgery | 23 |
| Majid O.W., 2013 | Use of dexamethasone to minimise post-operative sequelae after third molar surgery: Comparison of five different routes of administration | Oral Surgery | 22 |
| De Santana Santos T., 2011 | Evaluation of the muscle relaxant cyclobenzaprine after third-molar extraction | Journal of the American Dental Association | 21 |
| Trindade P.A.K., 2011 | Comparison of oral versus sublingual piroxicam during postoperative pain management after lower third molar extraction | International Journal of Oral and Maxillofacial Surgery | 20 |
